# Integrated Smoking Cessation for Smokers With Serious Mental Illness: Protocol for a Convergent Mixed Methods Implementation Evaluation Study

**DOI:** 10.2196/25390

**Published:** 2021-07-27

**Authors:** Kristina Schnitzer, Melissa Culhane Maravić, Diana Arntz, Nathaniel L Phillips, Gladys Pachas, A Eden Evins, Michael Fetters

**Affiliations:** 1 Center for Addiction Medicine Massachusetts General Hospital Boston, MA United States; 2 Harvard Medical School Boston, MA United States; 3 Mixed Methods Program University of Michigan Ann Arbor, MI United States; 4 Department of Family Medicine University of Michigan Ann Arbor, MI United States

**Keywords:** mixed methods implementation evaluation, pharmacotherapeutic smoking cessation aids, serious mental illness, smoking cessation, tobacco

## Abstract

**Background:**

Tobacco smoking is associated with significant morbidity and premature mortality in individuals with serious mental illness. A 2-year pragmatic clinical trial (PCORI PCS-1504-30472) that enrolled 1100 individuals with serious mental illness in the greater Boston area was conducted to test 2 interventions for tobacco cessation for individuals with serious mental illness: (1) academic detailing, which delivers education to primary care providers and highlights first-line pharmacotherapy for smoking cessation, and (2) provision of community health worker support to smoker participants. Implementing and scaling this intervention in other settings will require the systematic identification of barriers and facilitators, as well as the identification of relevant subgroups, effective and unique components, and setting-specific factors.

**Objective:**

This protocol outlines the proposed mixed methods evaluation of the pragmatic clinical trial to (1) identify barriers and facilitators to effective implementation of the interventions, (2) examine group differences among primary care physicians, and (3) identify barriers that stakeholders such as clinical, payor, and policy leaders would anticipate to impact the implementation of effective components of the intervention.

**Methods:**

Qualitative interviews will be conducted with all study community health workers and selected smoker participants, primary care providers, and other stakeholders. Measures of performance and engagement will guide purposive sampling. The Consolidated Framework for Implementation Research will guide qualitative data collection and analysis in accordance with the following framework approach: (1) familiarization, (2) identifying a thematic framework, (3) indexing, (4) charting, and (5) mapping and interpretation. Joint display analyses will be constructed to analyze and draw conclusions across the quantitative and qualitative data.

**Results:**

The 3-year cluster-randomized trial has concluded, and the analysis of primary outcomes is underway. Results from the pragmatic trial and this mixed methods implementation evaluation will be used to help disseminate, scale, and expand a systems intervention.

**Conclusions:**

The results of this mixed methods implementation evaluation will inform strategies for dissemination and solutions to potential barriers to the implementation of interventions from a smoking cessation trial for individuals with serious mental illness.

**International Registered Report Identifier (IRRID):**

DERR1-10.2196/25390

## Introduction

Tobacco smoking is associated with significant morbidity and premature mortality in individuals with serious mental illness. People with serious mental illness in the United States have physical diseases at a young age and die approximately 28 years earlier than those without mental illness, primarily from diseases that are directly attributable to tobacco smoking [1,2]. Premature mortality among individuals with serious mental illness is the largest lifespan disparity in the United States [3,4]. Recent estimates indicate that 64%-79% of individuals with schizophrenia spectrum disorders smoke tobacco regularly [5,6], as do 44%-71% of those with bipolar disorder [5-7] and 43% of those with major depressive disorder [8]. Most people with serious mental illness state that they would like to quit smoking but have not been offered smoking cessation treatment [9-11]. People with serious mental illness appear to be less likely to quit smoking [12] and more likely to experience relapse without pharmacotherapeutic smoking cessation aids [13], yet few are prescribed proven effective tobacco dependence pharmacotherapies together with behavioral treatment [14,15].

In a pragmatic clinical trial (PCORI PCS-1504-30472) conducted from November 2016 to February 2020, our research group tested the effectiveness of a 2-year intervention that involves (1) academic detailing, which involves the delivery of targeted smoking cessation education to primary care physicians and (2) community health worker support for tobacco abstinence among those with serious mental illness. Over 1100 smokers with serious mental illness—hereinafter referred to as “smoker participants”—enrolled in the trial in 2016. Primary quantitative analysis of study outcomes is underway.

Academic detailing is a provider-level educational intervention in the form of targeted, practical, action-oriented information, which is designed to educate providers on current best practices in a particular area. During this intervention, education is provided to primary care providers by doctoral-level (MD and PhD) study staff who are focused on the safety and tolerability of first-line pharmacotherapy for smoking cessation treatment in those with serious mental illness. The impact of academic detailing on the provision of smoking cessation pharmacotherapy to providers, and the resulting cessation outcomes, has not been studied.

Community health workers assist patients with accessing services and provide health education and outreach in their communities. Utilization of community health worker support has been demonstrated to be useful in reducing the length and number of hospitalizations, improving posthospitalization follow-up, and improving mental health in the general medical setting [16,17]. To date, interventions for community health workers have not been studied among those with serious mental illness or those specifically targeting smoking cessation. In this pragmatic clinical trial, we tested the provision of community health worker support to patients with a defined role and focused on promoting smoking cessation.

**Figure 1:**
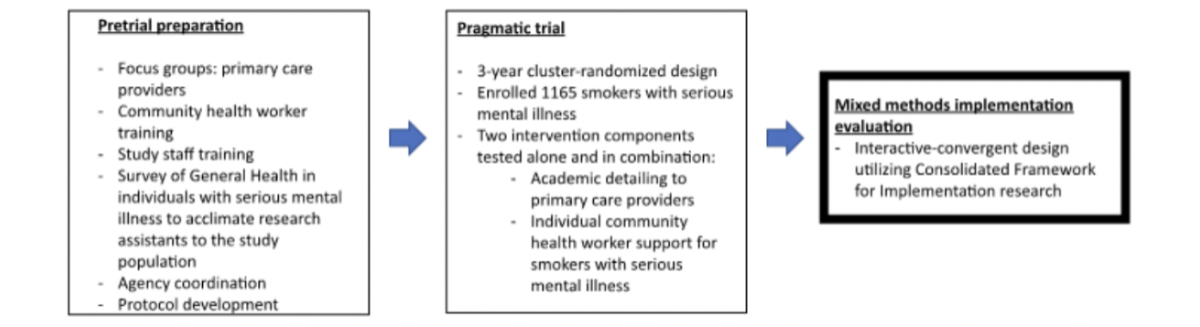
Overview of the pretrial preparation and the pragmatic trial leading to the mixed methods implementation evaluation in this study.

Figure 1 shows a procedural diagram of trial development, the trial, and the posttrial mixed methods implementation evaluation, the latter being the subject of this protocol.

Dissemination, scaling, and expansion of a systems intervention in a pragmatic clinical trial to other settings is greatly aided by the systematic identification of barriers and facilitators to implementation, and identification of relevant subgroups, effective and unique components, and setting-specific factors [18,19]. This mixed methods evaluation [20] will use categorical measures of engagement and performance (none or low, moderate, or high) to stratify subgroups of community health workers, smoker participants, and primary care providers to allow the analysis to be tailored and differentiated by group. Additionally, obtaining a deeper understanding of the 2 main intervention components, academic detailing and the community health worker services, will inform future health interventions for both those with serious mental illness and the general population. The complementary nature of quantitative and qualitative data will be leveraged to maximize our capacity to assess a broader range of contextual factors, generating data that are richer and more robust and actionable than either method alone [21]. This evaluation is additionally informed by implementation science, a field of methods and approaches that addresses challenges to the implementation of health interventions in usual practice settings [22].

We aim to conduct a mixed methods evaluation of the aforementioned pragmatic clinical trial to (1) identify barriers and facilitators to effective implementation of the interventions described by community health workers, smoker participants, and primary care providers in qualitative interviews; (2) examine how primary care providers, grouped at the clinic level, differ by quantitative performance and engagement level and how their experiences with the intervention components, barriers, and facilitators compare across these groups; and (3) identify barriers that stakeholders such as clinical, payor, and policy leaders would anticipate to impact the implementation of effective components of the smoking cessation intervention tested.

## Methods

### Design: Mixed Methods Intervention Evaluation Utilizing an Interactive Convergent Design

**Figure 2:**
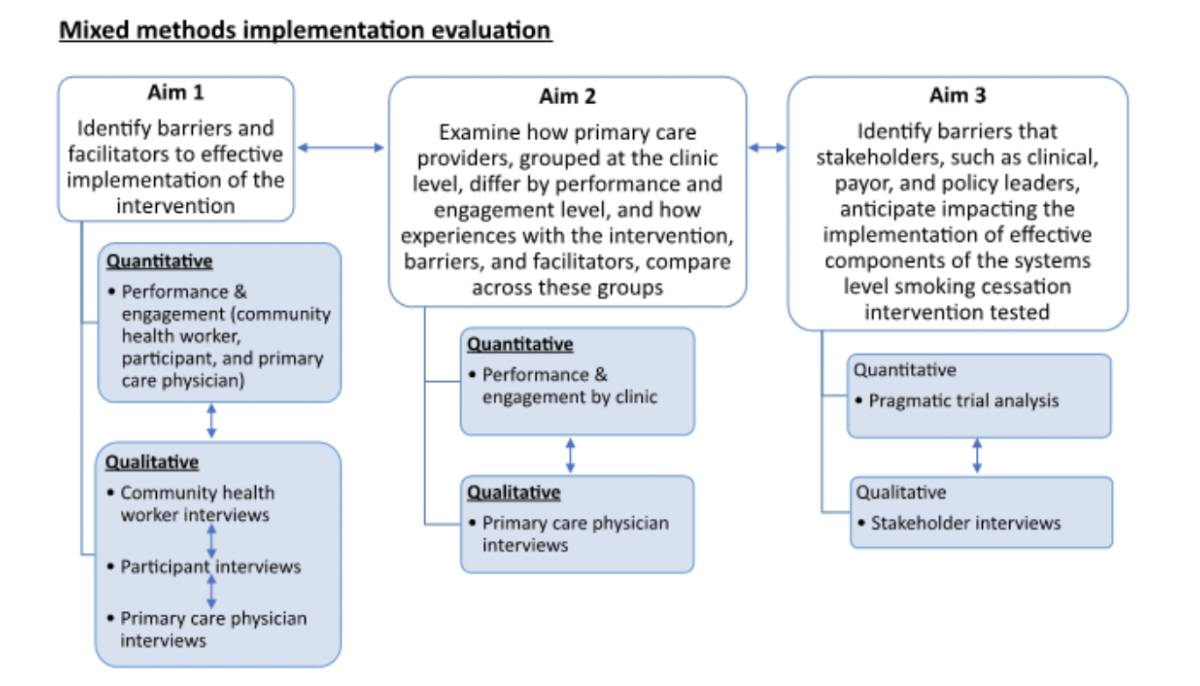
Mixed methods intervention evaluation utilizing interactive convergent design.

We propose a mixed methods evaluation [20] utilizing an interactive convergent design [23,24], which implies that qualitative and quantitative data are analyzed in tandem. As we code and analyze data, the emerging qualitative and quantitative findings will “talk” with each other so that the findings of each will inform additional analyses (Figure 2). This “iterative approach” denotes how the ongoing findings from each “strand”—that is, the qualitative or quantitative component of the inquiry in a mixed methods study [25]—will inform data collection of the other strand. We anticipate that findings from the qualitative data will yield hypotheses that will be examined quantitatively to further a comprehensive understanding of barriers and facilitators. Likewise, quantitatively derived findings will be enhanced by providing qualitative examples to potentially enhance the understanding of context and to validate the findings.

**Figure 3:**
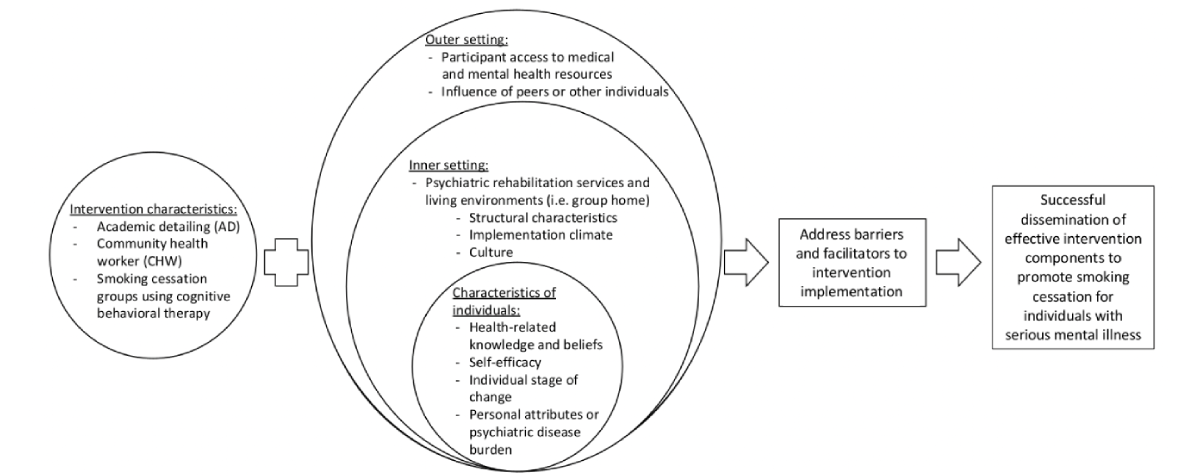
Logic diagram of the mixed methods intervention evaluation illustrating the structure of the Consolidated Framework for Implementation Research.

The Consolidated Framework for Implementation Research provides a basis for qualitative data collection and analysis [26]. This “meta-theoretical” framework, generated specifically for health care research, suggests that barriers are present at several levels, including organizational-level barriers (eg, lack of time and funds), provider-level barriers (eg, perceptions of academic detailing and the use of first-line pharmacotherapy in this population), and patient-level barriers (eg, stigma and knowledge of availability and accessibility resources and treatments). The Consolidated Framework for Implementation Research draws upon 19 different implementation models to create a typology of constructs to guide different phases of implementation studies and has been adapted for this study (Figure 3). Constructs of the Consolidated Framework for Implementation Research have been extensively utilized across settings and populations and provide a practical guide for the systematic assessment of barriers and facilitators when implementing interventions [26,27]. Specific variables that have previously been shown to impact the successful integration of evidence-based treatments and those that have been barriers to and facilitated future scale-up and dissemination strategies will be employed in this study [28,29].

### Quantitative Data

The quantitative data for this evaluation will be derived from the pragmatic clinical trial and process data from both interventions (ie, community health worker and academic detailing). Enrolled smoker participants were asked to complete three surveys: a baseline survey conducted at enrollment, followed by 2 surveys administered annually. Survey questions assessed self-reported current tobacco use, behaviors related to smoking cessation medications (eg, receipt of prescriptions from primary care physicians, prescription fills, and use of medications), smoking-related health issues, and rating of overall health. Demographic data were collected in the baseline survey. Additionally, smoker participants were asked to provide expired-air carbon monoxide measurements at each survey, which is used as biological confirmation of tobacco abstinence. Relevant process data include goal attainment during sessions with community health workers, the number of sessions with community health workers per smoker participant, attendance of the cessation group in cognitive behavioral therapy sessions, visits among primary care providers to address smoking cessation, and the attendance of primary care providers in academic detailing presentations.

Using these existing quantitative data, we anticipate creating matrices using 3-level measures (none or low, moderate, and high) of intervention engagement and performance to inform our purposive sampling for qualitative testing and future hypothesis testing. We will conduct quantitative analyses appropriate to establish thresholds for each measure of engagement and performance for community health workers, smoker participants, and primary care providers. We also anticipate conducting additional quantitative data analysis in testing hypotheses generated from the results of the qualitative data analysis.

### Qualitative Data

#### Eligibility, Setting, and Sampling

All community health workers paired with smoker participants in the trial (n=12) will be recruited to participate in qualitative interviews. Those eligible for qualitative interviews include smoker participants who provided consent to work with a community health worker and provided smoking behavior data at the year 2 survey (n=201). Primary care providers from clinics randomized to receive academic detailing, who treat smoker participants who provided information regarding prescriptions for smoking cessation medications at baseline and year 2 surveys, will be eligible to participate in the qualitative interviews (n=459). We intend to recruit stakeholder interviewees who are considered key opinion leaders in policy, payor, and clinical administration. Interviews will be conducted in person or in the community setting, depending on interviewee preferences.

Purposive sampling involves the intentional selection of study participants based on preselected criteria that relate to the research questions of interest; that is, barriers and facilitators. As described above, we will establish matrices based on 3-level categorical measures of engagement and performance, which will be used to frame our qualitative purposive sampling and ensure representation from a range of qualitative interview participants [29].

#### Interview Process

Basic concepts and the purpose of the study will be reviewed with all interviewees by a doctoral-level qualitative interviewer. Study staff will review the consent form with all interviewees and provide an opportunity to the participants to ask questions before signing the form. Additionally, smoker participants from the pragmatic clinical trial will be required to successfully complete a competency assessment in the form of a short quiz on facts about participation in the research qualitative interview. All individuals who complete the qualitative interview will receive US $15 as compensation for their participation.

Community health worker interviews will be conducted by authors DA, MCM, and KS, who are trained in qualitative interviewing techniques. Smoker participant interviews will be conducted by authors MCM and KS to minimize bias, as author DA interacted with many smoker participants during smoking cessation groups. Immediately following each qualitative interview, interviewers will document and organize field observations into 3 categories—“context, content, and concepts”—as described by Fetters and Rubenstein in 2019 [30]. This approach supports the integration of quantitative data (ie, inclusion of the number of smoker participant–completed community health worker or group visits in the “context” category), highlighting salient interview content and interviewer-identified important concepts, thus providing a foundation for future data integration. These field observations will be reviewed weekly throughout the data collection process to ensure that team members understand what information is emerging iteratively. This approach will allow for real-time adjustments in interview questions and provide the interviewer with an up-to-date sense of salient topics emerging from the interviews, which cannot be easily gleaned from a transcript.

#### Data Cleaning, Coding, and Analysis

All audio-recorded interviews will be transcribed verbatim. All transcripts will be checked for accuracy and completeness and edited where necessary. Final transcripts will be uploaded to NVivo software (version 12, QSR International) for organizing the text and supporting the analysis.

We will use a theory-driven approach in which we will explore the relationship between the findings and the Consolidated Framework for Implementation Research. Our study will be carried out in 5 stages outlined in the framework approach to qualitative analysis [31]: (1) familiarization, (2) identifying a thematic framework, (3) indexing, (4) charting, and (5) mapping and interpretation. Consolidated Framework for Implementation Research categories will form the basis for deductive analysis based on identified categories at the beginning of the study design, while new codes will be developed inductively by identifying those that emerge gradually from the data [32]. Based on the research questions, we will inductively identify themes and look for commonalities and variations in individual perspectives of the barriers and facilitators to study implementation. We will integrate quantitative and qualitative data for the purposes of convergence, contextualization, and expansion to gain a detailed understanding of processes and characteristics that can influence future scale-up and dissemination initiatives [30].

All analyses will follow procedures to ensure robust qualitative data analysis, including the establishment and training of the coding team and ongoing recalibration meetings to ensure reliability and to reduce coding drift. Interrater agreement will be assessed using a second rater coding a subset of interviews during initial data analysis until a high level of reliability (κ≥0.80) is established. Weekly consensus meetings will be held to resolve disagreements. The analytic process will be documented, and all coding decisions will be recorded for further review. Field observations will be reviewed to develop a macroscopic view of the data, assist the process of coding, and explore emerging hypotheses [31].

We will conduct joint displays analysis, in which quantitative and qualitative concepts will be merged and displayed together in an organized structure known as a “joint display” [33-35]. Combining quantitative and qualitative data in this way allows for broader interpretations across both types of data, so called “meta-inferences,” [29] to be drawn for the outcomes of interest, ideally achieving an integrated whole greater than a sum of the individual qualitative and quantitative aspects [24].

#### Modification: the COVID-19 Pandemic

The COVID-19 pandemic and resultant social distancing measures took effect during preparation for qualitative interviews. Consequently, the protocol has been adapted to allow for interviews to be conducted via telephone or videoconferencing. All in-person team meetings have been transitioned to videoconferencing platforms.

## Results

The 3-year cluster-randomized trial has concluded, and the analysis of primary outcomes is underway. Results from the pragmatic trial as well as this mixed methods implementation evaluation will be utilized to facilitate dissemination, scaling, and expansion of a systems intervention. The results of this evaluation will be published in a peer-reviewed journal and presented at scientific conferences.

## Discussion

This study aims to evaluate barriers and facilitators in implementing a smoking cessation intervention for individuals with serious mental illness. This study will also broadly evaluate the real-world implementation of academic detailing and community health workers in the role of smoking cessation and provide insights into engagement strategies and the smoking cessation process for individuals with serious mental illness. Through close examination of outcomes and particular challenges by subgroup, identification of effective and unique intervention components, and setting-specific factors in this study, our analysis will inform future health interventions for those with serious mental illness as well as the general population.
